# Colchicine Effectiveness and Safety in Periodic Fever, Aphthous Stomatitis, Pharyngitis, and Adenitis

**DOI:** 10.3389/fped.2021.759664

**Published:** 2021-11-25

**Authors:** Tatjana Welzel, Maren Ellinghaus, Anna L. Wildermuth, Norbert Deschner, Susanne M. Benseler, Jasmin B. Kuemmerle-Deschner

**Affiliations:** ^1^Pediatric Rheumatology and Autoinflammatory Reference Center Tübingen, University Children's Hospital Tübingen, University of Tübingen, Tübingen, Germany; ^2^Pediatric Pharmacology and Pharmacometrics, University Children's Hospital Basel (UKBB), University of Basel, Basel, Switzerland; ^3^Department of Anaesthesiology and Intensive Care Medicine, University Hospital Tübingen, University of Tübingen, Tübingen, Germany; ^4^Rheumatology, Department of Paediatrics, Alberta Children's Hospital, Cumming School of Medicine, Alberta Children's Hospital Research Institute, University of Calgary, Calgary, AB, Canada

**Keywords:** effectiveness, safety, disease activity, remission, outcome, PFAPA, corticosteroids

## Abstract

**Introduction:** Periodic fever, aphthous stomatitis, pharyngitis, and cervical adenitis (PFAPA) is the most common fever syndrome in childhood. High disease activity (DA) dramatically impacts the health-related quality of life. Thus, effective and safe treatment is crucial. Colchicine might be effective, but data are still lacking. Study aimed to assess colchicine safety and effectiveness in PFAPA.

**Methods:** This single center study was conducted between 03/2012 and 05/2021 in PFAPA patients without variants in genetic panel testing aged ≤ 18 years fulfilling Marshall criteria and classification criteria of Gattorno et al. Exclusion criteria were elevated liver enzymes, impaired kidney function, celiac disease, lactose intolerance, previous/ongoing biologics, known colchicine-intolerance. Demographics, clinical characteristics, treatment, DA, colchicine effectiveness and safety were recorded at baseline, first and last visit. Colchicine was started at 0.5–1.0 mg/day. DA was captured by physician (PGA) and patient/parent (PPGA) global assessment on a 10 cm visual analog scale, categorized as mild (<2), moderate (2–4), and high (≥5). Adverse event (AE) monitoring included gastrointestinal symptoms, liver enzyme/creatinine elevation, leukopenia, neutropenia. Primary outcome included response (R; composite of PPGA + PGA decrease ≥2) at last follow-up. Secondary outcomes were partial response (PR; PGA decrease = 1 + PPGA decrease ≥1), no response (NR; unchanged/worsened PGA/PPGA), colchicine safety, flare characteristics.

**Results:** Twenty-seven PFAPA patients were included, 52% were female, median age was 5.8 years (1–10.75), median follow-up time was 13 months. At baseline, median PPGA was high; median PGA moderate. All patients had febrile flares. Median flare frequency was every 4–5 weeks; median duration 5–6 days. Nine patients were pre-treated with corticosteroids, increasing flare frequency in 8/9. Primary Outcome: 17 patients (63%) were responders. Secondary outcomes: PR was achieved in 15%; NR in 22% at last follow-up. DA decreased significantly (*p* <0.0001). At last follow-up, 52% reported no flares, median flare duration decreased to 1–2 days. At first follow-up, 22% reported mild abdominal pain/diarrhea. Moderate abdominal pain/diarrhea occurred with ≥1 mg/day. Mild asymptomatic liver enzyme elevation or leucopenia were rare; no severe AE or colchicine discontinuation were observed.

**Conclusion:** Colchicine seems to be safe, well-tolerated, and effective in PFAPA patients. It can be considered in children with moderate/high DA even those without corticosteroid-benefit.

## Introduction

The periodic fever, aphthous stomatitis, pharyngitis and cervical adenitis (PFAPA) syndrome is one of the most common recurrent fever syndromes in children (1). The PFAPA syndrome belongs to the group of autoinflammatory diseases (AID) and is characterized by episodes of recurrent fevers lasting 3–6 days accompanied by characteristic oropharyngeal symptoms and cervical lymphadenitis in the absence of diarrhea, chest pain, skin rashes or arthritis (2). In the vast majority of cases, PFAPA has a good prognosis. It is self-limiting, typically in early adolescence and characterized by the absence of long-term sequelae (3). However, high inflammatory disease activity with frequently recurring flares impacts dramatically on the health-related quality of life and the well-being of the affected child and the whole family during the active disease phase in childhood (4, 5). Thus, effective control of disease activity, shortening of flares and reduction of flare frequency is crucial.

Recently the Childhood Arthritis and Rheumatology Research Alliance (CARRA) PFAPA working group has published consensus treatment plans (CTP) for the management of PFAPA including corticosteroids, colchicine, cimetidine and tonsillectomy (TE) (6). These CTPs need to be evaluated in future studies. Up to now, none of these treatment regimens has been approved for the management of PFAPA. In practice however, many children diagnosed with PFAPA are treated with corticosteroids, administered on demand in flares to abort disease symptoms. However, some of these patients do not respond favorably but rather experience an increase in disease activity including shortening of symptom-free intervals following corticosteroid administration (7, 8).

Colchicine is approved and highly effective in the management of Familial Mediterranean Fever (FMF) (9). Furthermore, there is data suggesting that colchicine might also be effective in children with PFAPA syndrome (10–13), including a randomized study with a total of 18 patients (11). However, there is limited evidence of colchicine effectiveness in PFAPA patients with moderate to high disease activity or increased disease activity after corticosteroid administration. Most importantly, data on colchicine safety in children with PFAPA is sparse. As the natural course of PFAPA is usually benign, data addressing safety of maintenance colchicine treatment enabling an evidence-based risk-benefit assessment are urgently needed. To support clinicians when caring for PFAPA patients, particularly those with moderate to high disease activity and non-beneficial corticosteroids treatment, data on colchicine safety and effectiveness are extremely valuable.

Therefore, the aim of this pilot study was: 1) to analyze the effectiveness of colchicine in PFAPA patients including children previously treated with corticosteroids, and 2) to report the safety of colchicine maintenance therapy in PFAPA patients.

## Materials and Methods

A single-center pilot retrospective cohort study of consecutive children diagnosed with PFAPA syndrome was performed between 03/2012 and 05/2021. Children and adolescents ≤ 18 years of age were included, after exclusion of other differential diagnoses if they showed normal growth and development. PFAPA was diagnosed based on 1) standardized assessments of recurrent disease flares with fever and typical PFAPA symptoms in the absence of other characteristic AID symptoms such as rash/erysipelas-like erythema, conjunctivitis, arthritis/arthralgia, diarrhea, peritonitis, pleurisy, and 2) accordance with classification criteria for PFAPA (2, 14). Furthermore, family history was screened for FMF. In addition, 3) the performed AID gene test panel had to be negative including pathogenic variants and variants of unknown significance as defined by the American College of Medical Genetics and Genomics (15). The AID gene test panel included the *MEFV, MVK, TNFRSF1A, NLRP3, NOD2, PSTPIP1, LPIN2, IL1RN, IL10RA, IL36RN* genes and was performed in a certified laboratory. Children were excluded, if they had 1) elevated liver enzymes or signs of impaired kidney function at time of study inclusion, 2) coeliac disease, lactose intolerance, evidence of irritable or chronic inflammatory bowel disease, 3) previous or ongoing biologic therapy, 4) evidence of Behçet disease according to the criteria for pediatric Behçet disease (16), or 5) known colchicine intolerance. Data was captured in the designated, institutional web-based Arthritis and Rheumatism Database and Information System (ARDIS) including standardized assessments of validated outcome measures at all visits (17). Study approval was obtained from the University of Tuebingen Institutional Review Board (012/2017BO2).

### Demographics, Clinical, and Laboratory Features

Demographic data included gender, age at AID diagnosis and at start of colchicine treatment. Furthermore, clinical symptoms at diagnosis, laboratory markers of inflammation during flares, previous therapies, and their effectiveness at time of referral to the study center were analyzed. PFAPA symptoms, flare frequency, flare duration and maximum temperature during flares were captured in a symptom diary modified from the Autoinflammatory Disease Activity Index (AIDAI) (18). Flare frequency was defined in categories from 0 to 4 with 0) no flares, 1) flares occurring less frequently than every 10 weeks, 2) occurring every 6–10 weeks, 3) every 4–5 weeks, and 4) every 3 weeks or less. Flare duration was defined in categories 0–3 with 0) no flares, 1) flares lasting 1–2 days, 2) 3–4, and 3) 5 or 6 days. Fever during flares was defined as a documented body temperature ≥38°C. Median temperature was calculated for all children with fever. At each visit, a complete physical examination including evaluation for disease activity and possible adverse colchicine events was performed and documented. Laboratory testing included monitoring of whole blood count, liver enzymes and kidney function. The study defined three distinct time points: 1) **baseline** defined as time of colchicine treatment start, 2) **first follow-up** defined as first visit after colchicine start (after 2–11 months), and 3) **last follow-up** defined as last study visit.

### Colchicine Therapy

Colchicine treatment was started at a dose of 0.5–1.0 mg/day. The therapeutic effect was monitored during visits. Based on disease activity and adverse events, colchicine dose was adjusted stepwise by 0.5 mg/day. During disease flares co-medication with non-steroidal anti-inflammatory drugs (NSAIDs) was permitted. Colchicine was discontinued if adverse events or intolerance occurred at the minimal dose of 0.5 mg/day. When the maximum tolerated colchicine dose did not result in improvement of disease activity or clinical symptoms, and PPGA and PGA were still high at follow-up visits, treatment change e.g., to Interleukin (IL)-1 inhibition or TE was considered.

### Definitions of Disease Activity and Response

Disease activity was captured by the physician global assessment (PGA) recorded on a 10 cm visual analog scale (VAS) with 0 representing no disease activity and 10 maximum disease activity. Disease activity was categorized as mild (PGA <2), moderate (PGA 2 ≤ 4), and high (PGA ≥ 5) at baseline, first and last follow-up. In addition, patient/parents‘ global assessment (PPGA) was measured and categorized similar to the PGA. Colchicine response was defined as response (R; PGA and PPGA decrease ≥ 2) and partial response (PR; PGA decrease = 1 and PPGA decrease ≥ 1). No response (NR) was defined as unchanged/worsened disease activity (PGA or PPGA).

### Definition of Colchicine Safety

Colchicine safety monitoring included assessment for abdominal pain and diarrhea; whole blood count, liver enzymes and kidney function. Abdominal pain/diarrhea was defined as “severe,” if symptoms were present daily, as “moderate” in case of being present three to six times per week and as “mild” when occurring one to two times per week. Liver enzyme monitoring included Alanine-Aminotransferase (ALAT), Aspartate-Aminotransferase (ASAT), Gamma-Glutamyltransferase (y-GT), Bilirubin and Lactate-Dehydrogenase (LDH). If the values of one or more of these enzymes were ≥10 U/L above the upper limit of the reference (ULR), the liver enzymes were defined as elevated. Serum creatinine increase was defined as values ≥10 mmol/l above the ULR. The ULRs are listed in Supplementary Table 1. Whole blood count was monitored to detect leukopenia which was defined as leukocyte count below 4,500/μl. In addition, the absolute neutrophile count (ANC) was assessed reflecting the percentage of neutrophils within the white blood count. ANC was classified as mild (1–1.5%), moderate (0.5– <1%) or as severe (<0.5%). Severe adverse events were defined as life threatening events, need for additional treatment, hospitalization or prolongation of hospitalization and they were recorded.

### Outcome

The primary outcome was R to colchicine at last follow-up. Secondary outcomes included 1) *overall colchicine responses*: (a) R, PR, and NR at first follow-up, (b) PR and NR at last follow-up, and (c) R, PR, NR for PFAPA patients previously treated with corticosteroids at first and last-follow-up; 2) *colchicine flare responses*: (a) flare frequency, (b) flare duration and (c) fever during flares and maximum temperature at first and last follow-up; 3) *colchicine safety*: (a) adverse events in general at first and last follow-up and (b) adverse events depending on colchicine dose at first and last follow-up, (c) severe adverse events, and (d) indication for treatment change or discontinuation.

### Analysis

Baseline demographics were analyzed using descriptive statistics; median values and ranges, mean values and interquartile ranges were computed. Comparative analyses were conducted using parametric and non-parametric methods as appropriate. R (version 3.5.1; R Development Core Team, Vienna, Austria, (http://r-project.org) was used for data analysis (PGA, PPGA) and visual graphics. Significances were tested using the Steel Dwass method. Decimals were rounded as appropriate.

## Results

A total of 27 patients were included in the study. Of these, 14 patients were girls (52%). The median age at PFAPA diagnosis was 3 years (range 1–9 years) (Table 1). At diagnosis, all patients had documented periodic febrile flares lasting 3–6 days accompanied by adenitis (100%), pharyngotonsillitis (96%), and aphthous stomatitis (44%) (Table 1). None of the patients had suggestive signs for FMF or FMF overlap including erysipelas-like erythema, rash, peritonitis, (chronic) arthritis/arthralgia or pleurisy. In addition, family history for FMF was negative. SAA and CRP were found to be highly elevated (SAA: mean 730 mg/L ± 272.6 with reference value <10 mg/L; CRP: 9.3 mg/dL ± 5.3 with reference values <0.5 mg/dL), when measured. Nine patients (33%) had received pre-treatment with corticosteroids resulting in control of PFAPA disease activity and shortening of flares in all patients (Table 1). However, in 8/9 patients (89%) the flare frequency increased dramatically; the symptom-free intervals shortened in the context of corticosteroid administration. All patients were treated symptomatically with NSAIDs during flares. In one patient a TE was performed, resulting in improvement but not in cessation of disease activity (Table 1).

**Table 1:** Baseline characteristics of PFAPA patients treated with colchicine.

**Characteristics**		**PFAPA cohort, *N* = 27**
**General characteristics**		
Female gender, *N* (%)		14 (52)
Age at diagnosis in years, median (range)		3.0 (1.0–9.0)
Age at baseline in years, median (range)		5.8 (1.0–10.75)
**Clinical symptoms**		
Recurrent periodic fevers, *N* (%)		27 (100)
Adenitis, *N* (%)		27 (100)
Pharyngotonsillitis, *N* (%)		26 (96)
Aphthous stomatitis, *N* (%)		12 (44)
Conjunctivitis, *N* (%)		0 (0)
(Chronic) arthritis or arthralgia		0 (0)
Diarrhea or peritonitis, *N* (%)		0 (0)
Rash including erysipelas-like erythema, *N* (%)		0 (0)
Pleurisy		0 (0)
**Previous therapies**		
Pre-treatment corticosteroids (*N* = 9)	Symptom termination during flare, *N* (%)	9 (100)
	Increased disease activity, *N* (%)	8 (89)
Pre-treatment tonsillectomy (*N* = 1)	Symptom improvement during flare, *N* (%)	1 (100)
	Increased disease activity, *N* (%)	0 (0)
**Flare characteristics**		
Flare duration in days, median (range)		5 (3–6)
Flare frequency in weeks, median (range)		4 (1–8)
**Last follow-up**		
Age at last follow-up in years, median (range)		7.1 (1.6–14.7)
Time baseline to last follow-up in months, median (range)		13.3 (5.0–66.5)

*PFAPA, periodic fever, aphthous stomatitis, pharyngitis, and adenitis; N, patients*.

### Colchicine Therapy

At baseline, the median age of the PFAPA cohort was 5.8 years (range 1–10.75 years, Table 1). The median time between baseline and first follow-up was 3.9 months (range 2–10.6 months) for the whole study group. When the two outlier patients with a visit after 2 months, and two outlier patients with delayed visits (8.5 and 10.6 months) were eliminated, the remaining patients had their first follow-up after a median of 3.9 months (range 3–5 months). The median time to last follow-up was 13.3 months (range 5–66.5 months). At baseline, the majority of patients (81%) were started at a colchicine dose of 0.5 mg daily; only five patients (19%) received 1 mg per day. The mean colchicine dose at first follow-up visit was 0.7 mg/day. At last follow-up, 10 patients (37%) received 0.5 mg colchicine daily, 12 patients (44%) 1 mg, three patients (11%) 1.5 mg, and two patients (7%) 2 mg colchicine per day.

### Disease Activity

At baseline, the overall physician-derived disease activity was moderate with a median PGA of 4 (range 1–6) (Table 2), including six patients (22%) with high disease activity (PGA ≥ 5) and one with mild disease activity (PGA <2). In contrast, patients/parents (PPGA) derived disease activity was high with a median PPGA of 5 (range 1–8) including 14 patients (52%) with high disease activity (PPGA ≥ 5) and two with mild disease activity (PGA <2) (Table 2). The corticosteroid pre-treated subgroup had comparable disease activity.

**Table 2:** Disease activity, flare characteristics, and colchicine effectiveness of 27 PFAPA patients treated with colchicine.

	**Baseline**	**First follow-up**	**Last follow-up**
**Disease activity**			
PGA (VAS in cm), median (range)	4 (1–6)	2 (0–4)	1 (0–4)
PPGA (VAS in cm), median (range)	5 (1–8)	2 (0–8)*	1 (0–8)
**Flare characteristics**			
Febrile flares, *N* (%)	27 (100)	18 (67)	11 (41)
Flare frequency category^a^ in weeks, median (range)	3 (2–4)	2 (0–4)	0 (0–4)
Flare duration category^b^ in days, median (range)	3 (2–3)	2 (0–3)	1 (0–2)
**Temperature**			
Temperature in °C, mean ± SD	40.3 ± 0.5	39.6 ± 0.7^Δ^	39.5 ± 1.1
**Colchicine effectiveness: complete PFAPA cohort**, ***N*** **(%)**			
Response		11/25 (44)	17/27 (63)
Partial response		8/25 (32)	4/27 (15)
No response		6/25 (24)	6/27 (22)
No flares anymore		7/27 (26)	14/27 (52)
Treatment discontinuation		0/27	0/27
**Colchicine effectiveness: pre-treated PFAPA patients with corticosteroids**, ***N*** **(%)**			
Response		6/8 (75)	5/9 (56)
Partial response		1/8 (13)	1/9 (11)
No response		1/8 (13)	3/9 (33)
No flares anymore		1/9 (11)	3/9 (33)
Treatment discontinuation		0/9	0/9

*PFAPA, periodic fever, aphthous stomatitis, pharyngitis and adenitis; PGA, physician global assessment; PPGA, patient/parent global assessment; VAS, visual analog scale.*

*^a^Frequency categories = 0: no flares; 1: > every 10 weeks; 2: every 6–10 weeks; 3: every 4–5 weeks, 4: every 1–3 weeks.*

*^b^Duration categories = 0: no flares; 1: 1–2 days, 2: 3–4 days, 3: 5–6 days.*

^*^Missing data from two patients.

*^Δ^Missing data from nine patients.*

*Response = PGA and PPGA decrease ≥ 2, Partial response = PGA decrease 1 and PPGA decrease ≥ 1, No response = unchanged disease activity (PGA or PPGA)*.

**Figure 1:**
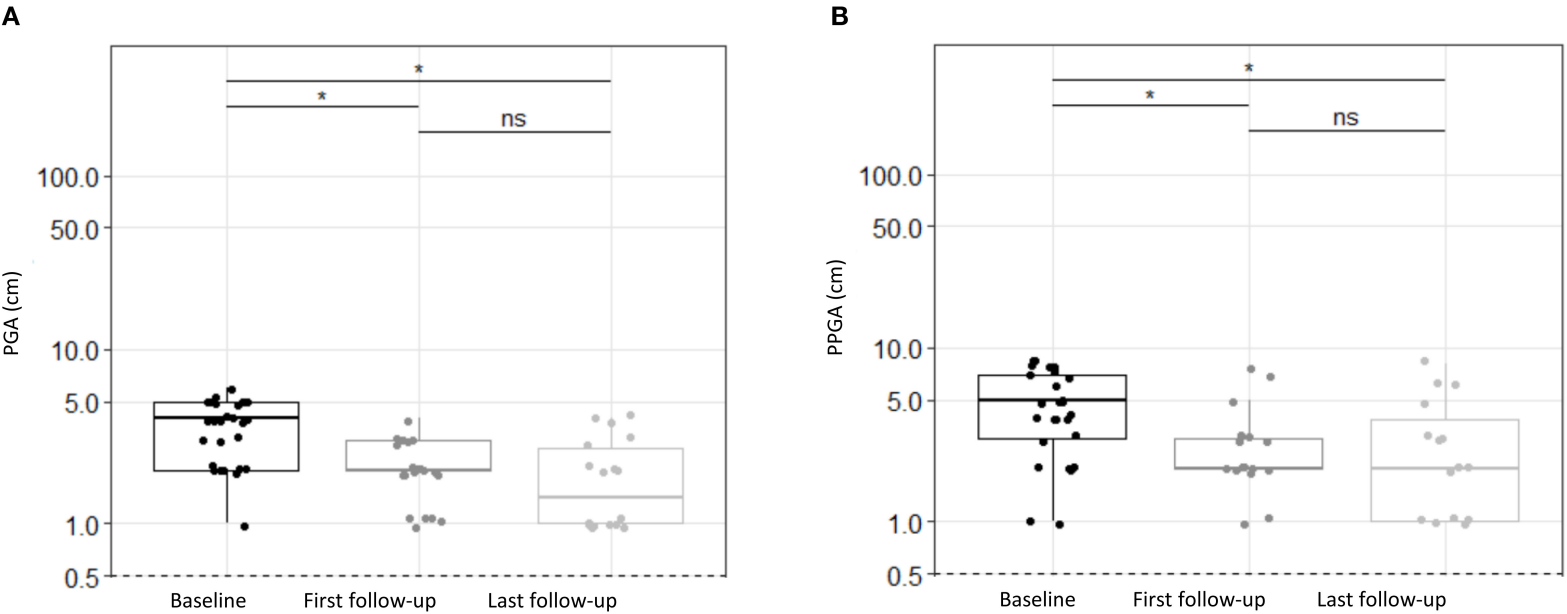
Disease activity estimated by physicians and patients/parents for PFAPA patients treated with colchicine. **(A)** Physician Global Assessment (PGA), **(B)** Patients/Parents Global Assessment (PPGA) depicted at baseline, first follow-up and at last follow-up. The values are presented at a log scale. Disease activity assessed as PGA or PPGA decreased significantly from baseline to first and last follow-up. There were no statistically significant changes (*p* > 0.05) between first and last follow-up. Significances were tested by Steel Dwass Methode. ns, not significant (*p* > 0.05), *p < 0.0001.

Colchicine treatment resulted in significantly decreased disease activity for the whole PFAPA cohort consistently documented by physicians (PGA) and patients/parents (PPGA). Disease activity decreased significantly according to PGA at first (*p* < 0.0001) and last follow-up (*p* < 0.0001) as well as according to PPGA at first (*p* = 0.0001) and last follow-up (*p* < 0.0001; see Figure 1; Table 2).

### Outcome

The primary outcome response (R) to colchicine was a combination of PGA and PPGA, mandating a decrease of ≥2 in both categories. At last follow-up, R was achieved in 17/27 patients (63%) (Table 2). The secondary outcomes included:

1) *Overall colchicine responses*: (a) At first follow-up, R was achieved in 11/25 patients (44%), PR in 8/25 patients (32%) and NR in six patients (24%) (Table 2). (b) At last follow-up, R increased as PR decreased. Six patients (22%) had NR (Table 2). (c) In the corticosteroid pre-treated subgroup, R was achieved in 5/9 patients (56%), PR in 1/9 patients (11%) and NR in three (33%) at last follow-up (Table 2). The patient not responsive to the TE, which was performed previous to study enrollment, showed complete response at first and last follow-up.
2) *Colchicine flare responses*: (a) Flare frequency improvement was documented in 24/27 patients (89%) at last follow-up. At baseline, the median flare frequency category was 3 (range 2–4) and decreased to 0 (range 0–4) at last follow-up (Table 2). At last follow-up 14/27 patients (52%) reported no flares (Table 2). (b) Flare duration was reduced in 25/27 patients (93%) at last follow-up., Median flare category at baseline was 3 (range 2–3) and decreased to 2 (range 0–3) at first follow-up and further to 1 (range 0–2) at last follow-up (Table 2). Of those with remaining flares, 5/27 patients improved to flare duration category 1 and 8/27 patients to category 2. (c) At baseline, all PFAPA patients (100%) had febrile episodes with a mean temperature of 40.3 ± 0.5°C (Table 2). At last follow-up, 11/27 patients (41%) reported febrile flares, with a mean temperature of 39.5 ± 1.1°C (Table 2). The patients not responsive to the TE had no flares anymore with colchicine 1 mg daily.
3) *Colchicine Safety*: (a) Adverse events were reported by 8/27 patients (30%) at first follow-up. Five patients had mild abdominal pain and diarrhea (one to two times per week), one had mild abdominal pain and two patients had mildly elevated liver enzymes (Table 3). No patient had elevated creatinine, leukopenia or abnormal ANC (Table 3). At last follow-up, 11/27 patients (41%) had adverse events (Table 3). Of these, five patients had reported adverse events at first follow-up. Adverse events at last follow-up are shown in Table 3. No abnormal ANC or creatinine values were documented. No severe adverse events were detected (Table 3). (b) Of the 8/27 patients (30%) reporting adverse events at first follow-up, six had received colchicine at 0.5 mg/day and two at 1 mg/day. In one patient with elevated liver enzymes, dose adjustment from 1 to 0.5 mg daily resulted in liver enzyme normalization. In two other patients, adverse events disappeared spontaneously without dose reduction. Five patients simply continued colchicine. Of the 11/27 patients (41%) reporting adverse events at last follow-up, one was treated with 0.5 mg/day, four with 1 mg/day, four with 1.5 mg/day and two patients with 2 mg daily. The patient receiving 0.5 mg/day had mild asymptomatic leukopenia. Patients treated with 1, 1.5, or 2 mg colchicine reported mild or moderate abdominal pain mainly in combination with diarrhea and one had mildly elevated liver enzymes without clinical symptoms (Table 4). Of note, three patients who had reported mild abdominal pain and mild diarrhea at first follow-up reported unchanged adverse event severity although colchicine was increased. (c) During this study no colchicine related severe adverse events were identified. (d) No colchicine discontinuation due to adverse events was necessary and no treatment change such as IL-1 inhibition or TE was indicated.

**Table 3:** Colchicine safety: adverse events of 27 PFAPA patients.

**Adverse event**	**Severity**	**First follow-up**	**Last follow-up**
Abdominal pain, *N* (%)	Mild^a^	6/27 (22)	5/27 (19)
	Moderate^b^	0	4/27 (15)
	Severe^c^	0	0
Diarrhea, *N* (%)	Mild^a^	5/27 (19)	3/27 (11)
	Moderate^b^	0	2/27 (7)
	Severe^c^	0	0
Liver enzyme elevation, *N* (%)	Mild	2/27 (7)	1/27 (4)
	Moderate	0	0
	Severe	0	0
Leukopenia, *N* (%)	Mild	0	2/27 (7)
	Moderate	0	0
	Severe	0	0
ANC decreased, *N* (%)	Mild	0	0
	Moderate	0	0
	Severe	0	0
Severe adverse events		0	0
**Response to adverse events**			
Treatment discontinuation, *N* (%)		0	0

*Severity grading: ^a^Abdominal pain and diarrhea: mild = one to two times/week, moderate = three to six times/week, severe = daily, ^b^liver enzyme elevation [modified according to (41)]: mild = ALAT/ASAT/LDH ≤ 3 ULR, BL/y-GT ≤ 1.5 ULR, moderate = ALAT/ASAT/LDH 3–5 ULR, BL/y-GT 1.5–3 ULR, severe = ALAT/ASAT/LDH >5 ULR, BL/y-GT >3 ULR, ^c^leucopenia [modified according to (42)]: mild ≥3,000 and <4,500/μL; moderate ≥2,000 and <3,000/μL; severe ≥1,000 and <2,000/μL, ANC: mild 1–1.5%, moderate 0.5– <1%, severe <0.5%.*

*N, patients; U, Units; L, liter; ULR, upper limit of reference; ALAT, Alanine-Aminotransferase; ASAT, Aspartate-Aminotransferase; y-GT, Gamma-Glutamyltransferase; LDH, Lactate-Dehydrogenase; BL, bilirubin*.

**Table 4:** Colchicine safety: adverse event dose relationship in 27 PFAPA patients.

**Colchicine dose**	**Patients, *N* (%)**	**Adverse event**	**Severity**
**First follow-up (adverse events in a total of 8 patients)**			
0.5 mg/day	5/27 (19)	Abdominal pain, diarrhea	Mild
	1/27 (4)	LDH elevated	Mild (ULR + 40 U/L)
1 mg/day	1/27 (4)	ALAT elevated	Mild (ULR + 20 U/L)
	1/27 (4)	Abdominal pain	Mild
**Last follow-up (adverse events in a total of 11 patients)**			
0.5 mg/day	1/27 (4)	Leucopenia	Mild (Leuc. 4,430/μl)
1 mg	1/27 (4)	Abdominal pain	Mild
	1/27 (4)	Abdominal pain, leucopenia	Mild (Leuc. 3,870/μl)
	1/27 (4)	Abdominal pain	Moderate
	1/27 (4)	Abdominal pain, diarrhea	Moderate
1.5 mg	1/27 (4)	LDH elevated	Mild (ULR + 20 U/L)
	1/27 (4)	Abdominal pain	Mild
	1/27 (4)	Abdominal pain, diarrhea	Mild to moderate
	1/27 (4)	Abdominal pain, diarrhea	Moderate
2 mg	2/27 (7)	Abdominal pain, diarrhea	Mild

*Severity grading: abdominal pain and diarrhea: mild = 1–2 times/week, moderate = 3–6 times/week; Liver enzyme elevation [modified according to (41)]: mild = ALAT/ASAT/LDH ≤ 3 ULR, BL/y-GT ≤ 1.5 ULR; Leukopenia: mild ≥3,000 and <4,500/μL.*

*PFAPA, periodic fever, aphthous stomatitis, pharyngitis and adenitis; mg, milligram; LDH, Lactate-Dehydrogenase; ALAT, Alanine-Aminotransferase; U, Units; L, Liter; ULR, upper limit of reference; BL, bilirubin*.

## Discussion

This study systematically evaluates the real-life colchicine effectiveness and safety in gene variant negative children with PFAPA syndrome. Importantly, it also included those patients with corticosteroid pre-treatment, of whom the vast majority had increased disease activity at baseline. Colchicine was found to be highly effective. Overall, disease activity decreased significantly (*p* < 0.0001). More than half of all PFAPA patients reported no flares anymore at last follow-up. Personalized colchicine dose adjustments during the study resulted in an additional increase in response. The safety profile of colchicine was favorable. Adverse events included abdominal pain, diarrhea, leukopenia and liver enzyme elevation. Overall, adverse events were reported as mild at first follow-up. Over time and with colchicine dose increase to ≥1 mg/day adverse events were more commonly reported with moderate severity. No severe adverse events were documented and no treatment discontinuation/change was necessary.

Colchicine was effective in PFAPA children with moderate to high disease activity and negative genetic testing, including those with increased disease activity/shortening of symptom-free intervals due to corticosteroids. Decrease of disease activity was statistically significant. The composite outcome of response indicated a response independently assessed by physicians and patients/parents. The median flare frequency category decreased from 3 (flares every 4–5 weeks) to 0 (no flares); those with ongoing flares reported a median flare duration of 1–2 days. Importantly, the vast majority of children, who experienced increased disease activity and/or shortening of symptom-free intervals after receiving corticosteroids for PFAPA had an excellent response to colchicine.

These data are in line with observations by Quintana-Ortega et al. (19), who suggested colchicine effectiveness in PFAPA patients with high disease activity and in those failing corticosteroid therapy. Colchicine treatment resulted in a decreased median flare duration from 4 to 1 days in 13 PFAPA patients (19). Butbul Aviel et al. performed a 6-month open label, randomized controlled study in 18 PFAPA patients. Of these, eight PFAPA patients were treated with colchicine (11). They observed a significant flare reduction in the colchicine treated patients compared to pre-treatment and compared to 10 untreated controls (11). Of note, in six colchicine treated patients (75%) *MEFV* gene variants were detected (11). Dusser et al. performed a retrospective multicenter study in 20 PFAPA patients treated with colchicine (12). Nine patients (45%) experienced a colchicine response. Response was defined as ≤ 2 febrile PFAPA flares in comparison to pre-treatment. Among those responder patients, 5/9 patients (71%) were heterozygous for *MEFV* gene variants. Therefore, Dusser et al. indicated that heterozygous *MEFV* gene variant carrier status tended to be more frequent in PFAPA patients with colchicine response. Similarly, Gunes et al. found that particularly in *MEFV* gene variant positive PFAPA patients, flare frequency was reduced due to colchicine treatment (13).

*MEFV* gene variants can affect not only treatment-response but may modify the clinical presentation. Heterozygous *MEFV* gene variant positive PFAPA patients (e.g., *M694V, E148Q, V726A*) may display significantly shorter and less periodic episodes (20, 21). Several patients may display FMF features, such as peritoneal/severe/excruciating or cramping abdominal pain, myalgia and arthralgia (22). Adrovic et al. suggested that although the newly proposed PFAPA criteria have satisfactory sensitivity, there may be a need for more distinctive criteria between PFAPA and FMF in FMF endemic regions (23). In 2019, the group proposed that symptoms of neck and head are commonly associated with PFAPA, while symptoms of the trunk and extremities are characteristic for FMF (24). In addition, Batu et al. suggested that Galectin-3 may be a promising biomarker to help in differentiating between PFAPA and FMF (25). Regarding treatment, both diseases have shown response to corticosteroids and colchicine (24).

Taken together, colchicine treatment should be considered for PFAPA patients with moderate to high disease activity, including those with increased disease activity/shortening of symptom-free intervals due to corticosteroids, even in the absence of *MEFV* gene variants as excellent effectiveness is consistently documented.

Long-term daily colchicine treatment was safe in the studied children with PFAPA syndrome. Adverse events included mild to moderate abdominal pain and diarrhea, mild liver enzyme elevation and mild leukopenia. Gastrointestinal symptoms were the most common adverse events, being only reported as moderate at colchicine doses of ≥1 mg daily. However, in general colchicine was well-tolerated at doses of 0.5–2 mg daily. In none of the PFAPA patients treatment discontinuation was necessary due to adverse events/severe adverse events.

These results are confirmed by Quintana-Oregana et al., who reported that in none of their 13 PFAPA patients colchicine discontinuation was required (19). Goldberg et al. studied colchicine treatment in FMF patients aged 0–8 years (26). They observed gastrointestinal symptoms as a common adverse event. Diarrhea occurred in approximately one fourth (24.4%) of patients <4 years and also in patients aged 4–8 years (22.9%) (26). This indicates a comparable safety profile of colchicine in small children and in older ones, assuring the use also in early childhood. Padeh et al. reported that 14.4% of FMF patients treated with colchicine developed diarrhea during a follow-up of 4 years (27). This is in line with the knowledge, that gastrointestinal symptoms are common in patients taking colchicine in recommended doses (28). Interestingly, Ferron et al. described a dose dependency of gastrointestinal symptoms in healthy volunteers exposed to colchicine (29). In addition to gastrointestinal complaints, liver enzyme elevation and hematologic abnormalities might be observed as rare or transitory adverse events. Padeh et al. reported a mild transitory increase of liver enzymes (45–158 IU/L) in 18 (11.8%) FMF patients during a follow-up period of 1 year, whereas blood cell counts and kidney function tests were normal (27). Goldberg et al. studied a total of 89 patients and observed a severe liver enzyme elevation (ASAT 172 U/L, ALAT 487 U/L) in one patient, mild transient neutropenia in another, and mild transient lymphopenia in a third patient (26). This highlights that bone marrow alterations due to maintenance colchicine treatment in therapeutic doses is rarely observed (30).

Colchicine is metabolized by cytochrome P (CYP) 3A4 and P-glycoprotein (PG). On the one side, individual over-expression of CYP3A4 and/or PG can result in lower colchicine response (31), on the other side drug-drug interactions (DDI) can lead to decreased colchicine metabolism with increased risk of adverse events, irrespective of normal kidney function (32). To avoid DDI, colchicine should not be combined with CYP3A4/PG-inhibitors. If these combinations are necessary, colchicine dose should be reduced (33). In addition, colchicine should be used cautious in patients with decreased kidney function (34). Similar to FMF-patients (9) regular laboratory monitoring of whole blood count, liver enzymes and kidney function should be performed in PFAPA children treated with colchicine.

In addition to corticosteroids and colchicine, TE is considered a therapeutic approach (35). Interestingly in a 2016 survey, rheumatologists were more likely to use colchicine compared to infectious disease physicians, who preferred TE (36). Overall, the risk of the medical treatment has to be weighed against that of surgery. The evidence for the effectiveness of TE in PFAPA is derived from two randomized controlled trials (37). In addition, 28 case series are reported indicating that TE may have a curative effect (38). Histology evidence suggests persistent inflammation in the absence of clinical symptoms in PFAPA tonsils, supporting the effectiveness of TE (39). Nevertheless, Yildiz et al. suggested that TE should be considered particularly in refractory cases after medical treatment (40).

Taken together, long-term colchicine seems to be a well-tolerated and safe treatment approach in PFAPA patients with moderate to high disease activity, particularly if DDI are avoided. The most common adverse events in this study were mild to moderate gastrointestinal symptoms, particularly in doses ≥1 mg.

This study has several limitations. The sample size of this study was small. However, the study aimed to include only clearly clinically defined PFAPA patients without any genetic variants. Tuebingen is a reference center, particularly providing support for patients with unclear phenotypes, comorbidities, organ damage and need for biological therapies. Due to a comprehensive clinical and genetic work-up, in several patients who are referred with suspected PFAPA syndrome, eventually another AID diagnosis is made. As follow-up study visits were included in clinical routine, the first follow-up time ranges between 2 and 10.6 months (median 3.9 months), as some patients with e.g., high disease activity were scheduled earlier, whereas other patients did not appear to their regular appointments and had to be re-scheduled. Therefore, if these four outliers are not considered, the remaining 23 patients (85%) had a homogeneous follow-up time ranging from 3 to 5 months with a median of 3.9 months. In addition, although this study was performed in a real-life cohort, there was only very few missing data. Importantly, based on the clear definition and comprehensiveness of outcomes and standardized outcome recording, the results are transparent and based on high- data quality. Even if, follow-up time for colchicine safety is limited, this study addresses well-defined adverse events. However, there is still a need for additional, ideally controlled treatment studies in PFAPA patients.

In conclusion, this study confirms that colchicine seems to be a safe and effective treatment approach for children with PFAPA syndrome and should be considered in patients with moderate to high disease activity and/or shortening of symptom-free intervals due to corticosteroids. Colchicine significantly decreased disease activity, reduced flare frequency, and shortened flare duration leading to increased quality of life for PFAPA patients and their families. Colchicine was safe and well-tolerated in the vast majority of PFAPA patients. No severe adverse events were observed. The most common adverse events were moderate gastrointestinal symptoms, only in doses ≥1 mg daily. Therefore, colchicine should be considered in children with mild to moderate PFAPA syndrome and negative gene variants even at low starting doses.

## Data Availability Statement

This dataset generated and analyzed during this study is not publicly available, but is available from the corresponding author on reasonable request after obtaining ethics approval. Requests to access these datasets should be directed to jasmin.kuemmerle-deschner@med.uni-tuebingen.de.

## Ethics Statement

The studies involving human participants were reviewed and approved by University of Tuebingen Institutional Review Board (012/2017BO2).

## Author Contributions

TW, ME, AW, ND, SB, and JK-D have contributed to the study design and conceptualization. AW, ME, and TW have been involved in data curation/data gathering. TW, ME, AW, ND, SB, and JK-D contributed in analysis. The original draft was prepared by TW and was reviewed and edited by ME, AW, ND, SB, and JK-D. SB and JK-D supervised the project. All authors have approved this version to be published, agreed to be accountable for all aspects in the work in ensuing questions related to the accuracy or integrity of any part of the work appropriately investigated and resolved, and agreed to the submission of this manuscript.

## Conflict of Interest

JK-D received grant support and speaker's fees from Novartis and SOBI. The remaining authors declare that the research was conducted in the absence of any commercial or financial relationships that could be construed as a potential conflict of interest.

## Publisher's Note

All claims expressed in this article are solely those of the authors and do not necessarily represent those of their affiliated organizations, or those of the publisher, the editors and the reviewers. Any product that may be evaluated in this article, or claim that may be made by its manufacturer, is not guaranteed or endorsed by the publisher.
