# Supplementary material for: Colchicine Effectiveness and Safety in Periodic Fever, Aphthous Stomatitis, Pharyngitis, and Adenitis
Source: Front Pediatr. 2021 Nov 25;9:759664. doi: 10.3389/fped.2021.759664 (PMC8655689; doi:10.3389/fped.2021.759664)
Supplement: Supplementary file 1 [file Table_1.pdf]

Supplementary Material

1 Supplementary Data

Table S1: Upper limit of reference

Laboratory value	Unit	Upper limit of reference
Aspartate-Aminotransferase (ASAT)	U/L	59
Alanine-Aminotransferase (ALAT)	U/L	39
Gamma-Glutamyltransferase (y-GT)	U/L	25
Bilirubin (BL)	mg/dl	1
Lactate-Dehydrogenase (LDH)	U/L	299
creatinine	mg/dl	0.6

Abbreviations: *U* units, *L* liters; *mg* milligram, *dl* deciliters
